# Post traumatic stress disorder and coping strategies among adult survivors of earthquake, Nepal

**DOI:** 10.1186/s12888-019-2090-y

**Published:** 2019-04-18

**Authors:** Ishwari Adhikari Baral, Bhagawati K.C

**Affiliations:** 1 Pokhara Nursing Campus, Tribhuvan University, Institute of Medicine, Pokhara, Nepal; 2 Gandaki Medical College Teaching Hospital and Research Centre, Tribhuvan University, Pokhara, Nepal

**Keywords:** Earthquake, PTSD, Coping strategies, Adult survivors

## Abstract

**Background:**

Post traumatic stress disorder (PTSD) is the most frequently reported psychiatric morbidity among the survivors of natural disasters. It is the main hindrance to rehabilitate their life. However its prevalence particularly in Nepal is largely unknown.

**Aims and objectives:**

To investigate the prevalence of post traumatic stress disorder and use of coping strategies among the adult survivors of earthquake.

**Methods:**

A cross- sectional descriptive study was carried out on a sample of 291 adult survivors after 10 months of Nepal Earthquake 2015. Study setting was Nuwakot district with multistage sampling (cluster sampling and systematic random sampling) method. PTSD checklist-5 was used to measure PTSD, and adapted and modified brief cope scale was used to assess coping strategies. Data were analyzed using descriptive and inferential statistics (independent t-test and one-way ANOVA) at 5% level of significance.

**Results:**

Study findings revealed that PTSD was prevalent among 24.10% of adult survivors with highest intrusion symptoms (3.24 ± 0.71). It was significantly associated with age (*p* = 0.017), sex (*p* = 0.013), education (*p* < 0.0001) and injury to self (*p* = 0.003). Elderly, females, illiterates and those who were injured during earthquake are at more risk for PTSD. Highest used coping strategy was active coping (2.92 ± 0.51). Survivors not having PTSD scored more on active coping (*p* < 0.0001) and self distraction coping (*p* = 0.006) while those with PTSD mostly used passive coping (*p* < 0.0001), religious coping (*p* < 0.0001) and substance use coping (*p* < 0.0001).

**Conclusion:**

Earthquake poses significant impact on mental health of the survivors. After 10 months of devastating earthquake, prevalence of PTSD among the survivors is high. Maladaptive coping strategies further increase possibility of PTSD. Effective screening and awareness program regarding promotion of positive coping strategies among the vulnerable groups should be reinforced for prevention of psychiatric morbidity among the survivors of earthquake.

## Background

On 25 April 2015, a huge earthquake measuring 7.8 Ritcher scale was experienced in Nepal with epicenter in Barpak Village Development Committee (VDC) of Gorkha district, 80 km northwest of Kathmandu. Another big aftershock of 7.3 magnitude again hit Nepal on 12^th^May, 2015. These two major earthquakes caused massive loss of lives and infrastructure in many districts [1]. As the consequence, 8702 people died while 22,493 were seriously injured [2]. A disaster disrupts the normal condition of existence and cause suffering that exceeds the capacity of adjustment of the affected community. It also imposes short and long term impacts on ecological, political, economic, developmental, social, physical and psychological dimensions [3]. While physical problems are addressed consequently, many of the mental problems remain undetected [4].

Trauma after earthquake is experienced by more than two thirds of general population at some point in their life resulting in wide range of mental and physical health consequences [5]. Studies suggest that 50% or more of the victims may develop chronic depression, pervasive anxiety and post-traumatic stress disorder (PTSD) which may cause long lasting suffering, disability and loss of income [6]. Post traumatic stress disorder has been found to be the most prevalent psychiatric morbidity after disaster [7]. Several study findings reveal higher burden of PTSD after natural disasters. According to systematic review on mental health and psychosocial consequences of natural disasters in Southeast Asia, the reported rates of PTSD symptoms following natural disasters range widely from 8.6 to 57.3% [4]. Determinants of PTSD are physical injury, property destruction, death in family, older age, female gender, low educational level and lack of social support [8–11]. Earthquake affected survivors try to cope with trauma in different ways. The adaptive mechanisms include use of religious, family and social support, exerting self-distraction and helping others. The less adaptive coping mechanisms that may need interventions include expression of stress in somatic form, denial, avoidance, blaming, helplessness, dependency and substance use. Maladaptive coping strategies further increase survivors’ vulnerability to PTSD [6].

The victims of earthquake undergo tremendous psychological consequences which largely depend upon the coping abilities of the survivors. The issue of psychiatric morbidity after the earthquake needs to be explored as early as the physical problems in order to promote positive mental health among the earthquake affected victims [4]. In developing countries like Nepal, which is in high risk for natural disasters, many of the emotional and mental health problems after disaster go unrecognized leading to long term psychiatric morbidity. Thus present study aims to

- assess the prevalence of PTSD among the adult survivors of earthquake.
- identify the coping strategies used by adult survivors of earthquake.
- find out the association between PTSD and socio-demographic and earthquake related variables.
- compare difference in subscale scores of coping strategies according to socio-demographic variables and PTSD status.

## Methods

A descriptive cross sectional research design was used to assess the posttraumatic stress disorder and coping strategies among the adult survivors of Nepal earthquake 2015. Data was collected after 10 months of earthquake at Nuwakot district which was one of the most affected districts of Nepal in 2015 earthquake with 93.24% of households fully damaged [Government of Nepal] [12].

Sample size was 291 based on sample size estimation formula [Sample size (n) = Z^2^pq/l^2^] where, *p* = 12.7% (prevalence of adult survivors of earthquake in Tamil Nadu India) [4]. Thus, sample size was 4 × 0.127 × 0.873/0.0025 = 177. Design effect 1.5 was taken for cluster random sampling (177 × 1.5 = 265). Adding 10% for non-response rate, the final sample size was 291.

Multistage sampling technique was used to select the earthquake affected households of Nuwakot, Nepal. At first cluster random sampling was used to select three wards from three selected VDCs of Nuwakot. Then the sample number was determined by proportionate allocation according to the number of households in each selected ward. Finally, samples were selected from the households by using systematic random sampling. At the household, where more than one affected adult were present, random selection was done by lottery method. Only the adult survivors of age 20 years and above who had experienced the earthquake in Nepal on April 25, 2015 were included in the study.

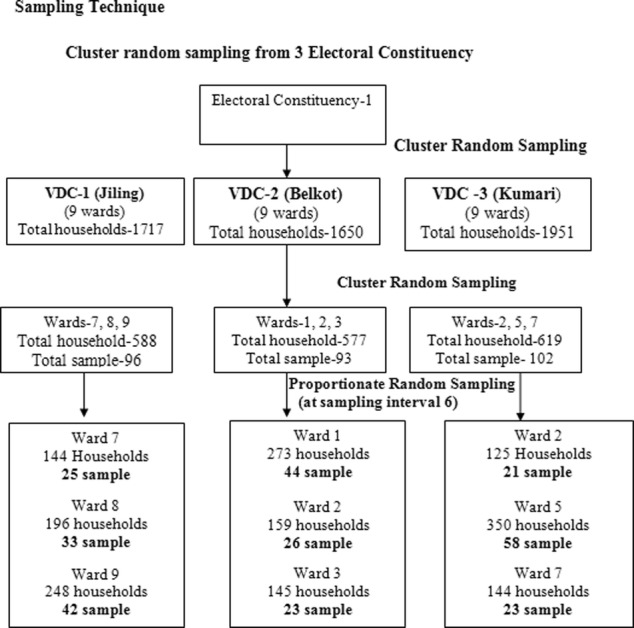



### Data collection procedure

Data were collected by using Nepali version, pre-tested interviewer administered questionnaire. Questions regarding socio-demographic characteristics and earthquake related characteristics were developed by the researcher herself specifically for this study after an extensive literature review on the similar subject matter. PTSD was measured by standard PTSD symptoms checklist according to DSM-5 criteria (PCL-5) developed by National Centre for PTSD [13]. It is a self report rating scale for measuring PTSD caused by traumatic event and comprises 20 items. Coping strategies were measured by brief cope scale developed by Malik [14] to use for earthquake survivors of Kashmir earthquake, 2005. The instrument consists of 41 items further sub grouped into six subscales representing specific coping styles namely religious coping, passive coping, active coping, social coping, substance use coping and self distraction coping.

Validity and reliability of both tools have been documented [14]. PCL-5 is a psychometrically sound measure of PTSD symptoms and PCL-5 scores exhibited strong internal consistency (α = 0.94) and test-retest reliability (*r* = 0.82) [15]. All the items of brief cope scale are related to coping measures used after earthquake. The chronbach’s alpha coefficient for the total scale was 0.83. Internal consistency for the cope subscales were religious coping: (*r* = 0.91), passive coping: (*r* = 0.85), active coping: (*r* = 0.84), social coping: (*r* = 0.73), substance use coping: (*r* = 0.91), self distraction coping: (*r* = 0.57) [14].

For the use of tool in Nepalese context, first forward translation (from English to Nepali) and then backward translation (Nepali to English) was done by two independent bilingual translators. To identify accuracy, clarity and consistency of the tool, pretesting of the tool was conducted on 29 samples in the similar setting and necessary modification was done. Chronbach’s alpha value of PCL-5 checklist was 0.89 and that for cope scale was 0.79 showing higher degree of internal consistency of the tool.

Data was collected at the place convenient for respondents in their residence by face to face interview. Informed verbal and written consent was obtained from the respondents prior to the data collection with information about the nature of the study and their role in research. They were also informed about the purpose of the study, their voluntariness in participation and no any foreseeable risk and harm in the study. The average time taken for the questionnaire was about 20–30 min. About 12–14 respondents were interviewed in a day and data was collected from March 3, 2016 to March 26, 2016.

### Statistical analysis

Data were analyzed using statistical Package for Social Science 16 (SPSS 16). For PTSD checklist, total score was obtained by summing up the responses of each item. Then the participants with a score of 33 or higher were classified as having PTSD, and participants with a score lower than 33 as not having PTSD. This classification was done according to the criteria given by National Centre for PTSD [13]. For coping scale, analysis was performed by obtaining the sum as well as mean value of each subscale. The mean score was obtained by adding the score of all items as well as each item of all subscales in order to attain a comparable ture. Data were interpreted as higher the mean score, higher the use of specific coping style.

The normality test of the data revealed normal distribution of the data. In descriptive statistics, frequencies, percentages, mean and standard deviations were calculated. For inferential statistics, chi-square test was used to assess association between PTSD and socio-demographic variables and earthquake related variables. The difference in scores of each subscale of coping strategies in relation to different socio-demographic variables and PTSD status of the adult survivors was examined by independent *t*-test and one way ANOVA. The level of significance was considered at 5% with *p* value < 0.05and 95% confidence interval.

## Results

### Characteristics of the respondents

Tables 1 and 2 shows the socio-demographic and earthquake related characteristics of the adult survivors.

**Table 1:** Socio-demographic characteristics of the respondents

*n* = 291		
Characteristics	Frequency	Percentage
Age group^a^ in Years(*n* = 291)		
20–39	127	43.64
40–59	113	38.83
60 and more	51	17.53
mean age: 43.60 ± 14.40; (Min-20; Max-82)		
Sex (*n* = 291)		
Male	166	57.04
Female	125	43.06
Ethnicity/Caste (*n* = 291)		
Brahmin/ Chettri	172	59.11
Janajati	94	32.29
Dalit	25	8.60
Religion (*n* = 291)		
Hindu	234	80.41
Buddhist	44	15.12
Christian	13	4.47
Marital status (*n* = 291)		
Married	255	87.63
Unmarried	26	8.93
Widow/Widower	10	3.44
Educational Status (*n* = 291)		
Illiterate	147	50.52
Literate	144	49.48
Among the Literate^b^, (*n* = 144)		
Can read and write only	35	24.30
Basic (grade 1–8)	27	18.75
Secondary (grade 9–12)	63	43.75
Higher level bachelor and above)	19	13.20
Occupation (*n* = 291)		
Farming	172	59.11
Homemaker	55	18.90
Service holder	26	8.93
Student	21	7.22
Business	13	4.47
Laborer	4	1.37
Living Arrangement (*n* = 291)		
Living in temporary home	245	84.20
Living in own home	46	15.80

^a^Age group according to Erik Erikson Developmental stage

^b^Literacy category according to Ministry of Education(MOE) Nepal, 2013

**Table 2:** Earthquake -related characteristics of the adult survivors

*n* = 291		
Characteristics	Frequency	Percentage
Injury to respondent (*n* = 291)		
Injured	7	2.41
Not injured	284	97.59
Injury to family members (*n* = 291)		
Injured	14	4.81
Not injured	277	95.19
Extent of damage to house (*n* = 291)		
Partially damaged	20	6.87
Fully damaged	271	93.13
Loss/ damage to property (*n* = 291)		
Lost/damaged	62	21.30
No loss/damage	229	78.70

### Socio-demographic and earthquake related characteristics of survivors

Overall mean age of adult survivors was 43.60 ± 14.40 ranging from 20 to 82 years. Fifty seven percent were male. About 60% were Brahmin/Chettri followed by Janajati (32.29%). Half (50.52%) of them were illiterate. Majority (84.20%) were living in temporary home after earthquake (Table 1). Findings also depict that 2.41% of survivors were injured while 97.59% had no any injury. Moreover, 4.81% reported injury to their family members. Majority (93.13%) of their houses were fully damaged requiring full repair. Loss or damage to their property was reported by 21.30% survivors (Table 2).

### Prevalence of PTSD and coping strategies used by the respondents

The overall mean score on PTSD checklist was 25.80 ± 14.75 (min-0; max-68). Among the survivors, 24.10% met the criteria for PTSD (Table 3). The intrusion symptoms were high among the survivors with PTSD with mean score 3.24 ± 0.71 (Table 4). The mean score of overall cope scale was 2.30 ± 0.28. Survivors’ mean score was highest on active coping (2.92 ± 0.51) strategy followed by social coping (2.69 ± 0.52). Substance use coping was less frequently used coping strategy with mean score (1.55 ± 0.67) (Table 5).

**Table 3:** Prevalence of PTSD

PTSD status	Frequency	Percentage
PTSD Present (PCL-5 score 33 and above)	70	24.10
PTSD Absent (PCL-5 score less than 33)	221	75.90
Total	291	100.00

Overall mean score on PTSD checklist: 25.80 ± 14.75 (min-0; max-68)

**Table 4:** PTSD symptoms score among the respondents

Domains of PTSD	Mean	S.D	S.E	Confidence interval
Intrusion Symptoms	3.24	0.71	0.08	(3.07, 3.41)
Avoidance of Stimuli	1.29	0.89	0.10	(1.08, 1.50)
Negative alteration in cognition and mood	2.16	0.70	0.08	(1.99, 2.33)
Alteration in arousal and reactivity	2.13	0.60	0.07	(1.98, 2.27)

**Table 5:** Coping Strategies score among the respondents

Coping strategies	Mean	S.D	S.E	Confidence interval
Overall Cope score	2.30	0.28	0.02	(2.26, 2.33)
Religious Coping	2.35	0.67	0.04	(2.27, 2.43)
Passive coping	1.70	0.64	0.04	(1.63, 1.78)
Active coping	2.92	0.51	0.03	(2.86, 2.97)
Social coping	2.69	0.52	0.03	(2.63, 2.75)
Self distraction coping	1.86	0.76	0.04	(1.77, 1.95)
Substance abuse coping	1.55	0.67	0.04	(1.47, 1.63)

### Association between respondent’s characteristics and PTSD

As shown in Table 6, significant statistical association was observed for age (*p* = 0.017), sex (*p* = 0.013) and educational status (*p* < 0.0001) of the respondents. However, no significant association was observed for living arrangement whether the survivors are living in own home or temporary home (Table 6). PTSD was significantly higher among the survivors who were injured because of earthquake (*p* = 0.003). Conversely, there was no significant association for injury to family members, extent of damage to house and loss or damage to property (Table 7).

**Table 6:** Association of PTSD with socio-demographic variables

Variables	PTSD		*p*- value
	Present frequency (%)	Absent frequency (%)	
Age			
20–39	22 (17.3)	105 (82.7)	0.017*
40–59	29 (25.7)	84 (74.3)	
≥ 60	19 (62.7)	32 (37.3)	
Sex			
Male	31 (18.7)	135 (81.3)	0.013*
Female	39 (31.2)	86 (68.8)	
Educational Status			
Illiterate	65 (25.5)	190 (74.5)	< 0.0001*
Literate	5 (13.9)	31 (86.1)	
Living Arrangement			
Living in own home	14 (30.4)	32 (69.6)	
Living in temporary home	56 (22.9)	189 (77.1)	0.270

*p value significant at < 0.05 level of significance

**Table 7:** Association of PTSD with Earthquake-related Variables

Variables	PTSD		*p*- value
	Present frequency (%)	Absent frequency (%)	
Injury to self			
Injured	5 (71.4)	2 (28.6)	0.003*
Not injured	65 (22.9)	219 (77.1)	
Injury to family members			
Injured	3 (21.4)	11 (78.6)	0.814
Not injured	67 (24.2)	210 (75.8)	
Damage to house			
Partially damaged	4 (20.0)	16 (80.0)	0.660
Fully damaged	66 (24.4)	205 (75.9)	
Loss/damage to property			
lost/damaged	20 (28.6)	42 (71.4)	0.088
No loss/damage	50 (19.0)	179 (81.0)	

*p value significant at < 0.05 level of significance

### Association between respondent’s characteristics and coping strategies

Study findings revealed that there was difference in religious coping score according to age (*p* < 0.0001), sex (*p* = 0.033) and educational status (*p* < 0.0001) of the adult survivors (Table 8). Religious coping was found to be significantly high in elderly adults and illiterate survivors compared to their counterparts. Significant difference in passive coping was found according to sex (*p* < 0.0001) and educational status (*p* < 0.0001) of the survivors. Passive coping was higher in females and among the illiterates. Conversely, active coping was significantly higher among the younger adults (*p* = 0.022), males (*p* < 0.0001) and the literate (*p* < 0.0001). Moreover, self distraction coping score was significantly higher among young adults (*p* < 0.0001), males (*p* = 0.033) and literates (*p* < 0.0001). Significant difference was observed between substance use coping with age (*p* < 0.0001) and educational status (*p* < 0.0001) of respondents. It was higher among elderly adults and those survivors who were illiterate (Table 8).

**Table 8:** Association between respondent’s characteristics and coping strategies

Demographic variables	Coping strategies (mean scores)					
	RC	PC	AC	SC	SDC	SUC
Age						
20–39	2.09	1.66	3.00	2.64	2.30	1.35
40–59	2.42	1.71	2.89	2.73	1.54	1.64
≥ 60	2.85	1.80	2.70	2.75	1.50	1.90
^a^*p* value	< 0.0001*	0.381	0.022*	0.258	< 0.0001*	< 0.0001*
Sex						
Male	2.28	1.52	3.02	2.72	1.94	1.61
Female	2.45	1.95	2.78	2.58	1.75	1.48
^b^*p* value	0.033*	< 0.0001*	< 0.0001*	0.075	0.033*	0.092
Educational Status						
Illiterate	2.64	1.90	2.78	2.68	1.50	1.77
Literate	2.05	1.51	3.06	2.70	2.23	1.33
^b^*p* value	< 0.0001*	< 0.0001*	< 0.0001*	0.716	< 0.0001*	< 0.0001*

Higher score indicates higher coping mechanism

*RC* Religious coping, *PC* Passive coping, *AC* Active coping, *SC* Social coping, *SDC* Self Distraction Coping, *SUC* Substance Use Coping

^a^anova test

^b^t-test

*p value significant at ≤0.05 level of significance

### Coping strategies according to PTSD status

Respondents having PTSD had significantly higher score on religious coping, passive coping, self distraction coping and substance use coping and on the other hand survivors with no PTSD had significantly higher scores on active coping (Table 9).

**Table 9:** Coping strategies according to PTSD status

PTSD status	Coping strategies (mean scores)					
	RC	PC	AC	SC	SDC	SUC
Present	2.73	2.33	2.70	2.61	2.65	1.86
Absent	2.24	1.50	2.98	2.72	1.93	1.46
*p*-value	< 0.0001*	< 0.0001*	< 0.0001*	0.135	0.006*	< 0.0001*

Higher score indicates higher coping mechanism

*RC* Religious coping, *PC* Passive coping, *AC* Active coping, *SC* Social coping, *SDC* Self Distraction Coping, *SUC* Substance Use Coping

*p significant at ≤0.05 level of significance

## Discussion

The primary finding of current study reveals that 10 months after a devastating earthquake, PTSD is prevalent among 24.10% of the earthquake affected adult survivors. Thus PTSD symptoms are experienced by the earthquake affected survivors even almost a year after the traumatic event and need specific diagnosis and intervention in order to prevent long term psychiatric morbidity.

The study findings highlight that PTSD was significantly associated with age of the adult survivors of earthquake. It was present in more than half (62.7%) of the elderly adult survivors. This finding is in agreement with studies conducted in China and Italy [11, 15]. The findings imply that the risk of PTSD increases with age. This vulnerability can be attributed to physical impairment, chronic health condition, low coping level and decreased adaptation to stress among the elderly adults [15].

Gender difference in prevalence of PTSD was observed in this study. PTSD was present in (31.2%) of the total female survivors whereas only 18.7% of total male survivors met criteria for PTSD. We can infer that females are more vulnerable for PTSD than males. Consistent finding was revealed by almost all other evidences of PTSD in relation to gender [8, 16–19]. A study by Shrestha [20] after Nepal Earthquake, 2015 also reported higher PTSD among female medical personnel. Higher PTSD risk among women may be due to their stronger perceptions of threat and loss of control. Females also have perception of insufficient social support resources. Literature even explains gender differences in neuro-endocrine response that leads to higher risk of PTSD in women [21].

The prevalence of PTSD was only 13.9% among the literate survivors. Conversely, illiterate accounted for 38.1% prevalence. Higher educational level enhances the individual’s trauma understanding ability, which could improve confidence in physical as well as mental health recovery thus preventing the stress disorder. Many other studies also reported low educational attainment as a risk factor for development of PTSD after natural disaster [5, 8, 19]. Study findings also revealed that having being injured as the significant risk factor for post earthquake PTSD which is compatible with findings of other studies [5, 11].

### Use of coping strategies by adult survivors

Among the overall coping strategies used by the adult survivors of earthquake, the highest used coping strategies was active coping (2.92 ± 0.51) followed by social coping (2.69 ± 0.52). Conversely, lowest used coping strategy was substance abuse coping with mean score (1.55 ± 0.67). This finding is in line with the study done in Italy [18]. A possible interpretation of this finding could be that adults though largely affected by earthquake, more frequently adopted positive coping strategies by actively involving in changing their circumstances and problem-solving activities and socializing more than before [22].

Furthermore, average survivors also utilized religious coping practice like praying and worshiping, believing in god’s help, doing charity in the name of god as their strategy to overcome the stress. This may be possibly because Nepalese are religious with strong belief on god’s help at both good and bad times. Moreover, people regard natural disasters as ‘act of god’ and practice religious rituals in order to please god so that this traumatic event would not occur in future. This corresponds to the findings by many studies revealing use of religious coping for their positive adjustment after the earthquake [8, 10, 23].

### Relationship of subscale score of coping strategies with socio-demographic variables

Study findings depicted that elderly adults were more devoted towards religious coping practice compared to their counterparts. This implies that elderly people believe that god always help people at good or bad time. Young and middle aged adults scored higher in active coping and self distraction coping. A study from Italy also suggested consistent findings [18].

Gender difference was also noted in use of different coping strategies by the earthquake affected individuals. Female adult survivors were more devoted towards religious coping and passive coping whereas male survivors more frequently used active coping, social coping and self distraction coping. Evidences also revealed gender differences in use of coping styles [10, 24, 25]. Possible interpretation for this finding might be that men are more likely to deal with the event by deemphasizing what has happened and actively facing the situation to solve their problems. Women, by contrast, are more likely to deny what happened to them and being passive to keeping themselves away from dealing with the situation [26]. Illiterate adult survivors more frequently used religious and passive strategies whereas literates most frequently used active coping and self distraction coping. This finding is consistent with the study conducted by Roohafza et al. [27].

### Relationship of coping strategies with PTSD status of adult survivors

The study findings reveal that adult survivors with PTSD often employed maladaptive coping strategies for dealing with earthquake and had significantly higher scores in the domains of passive coping and substance use coping. Religious coping was also higher among those with PTSD. In contrast, survivors without PTSD generally employed adaptive coping mechanisms like active coping and self distraction coping strategies. Thus, the presence of adaptive coping in survivors who did not develop PTSD after the earthquake and maladaptive coping in those who manifested disorder suggests the existence of a relationship between the two variables. Previous studies also reveal similar findings [18, 22].

### Limitations

Present study was conducted only among the adult earthquake survivors of Nuwakot district during 4 weeks of data collection period thus it may not represent the whole survivors of earthquake in Nepal, 2015.This study used only the cut off score of self report PCL-5 checklist without considering the DSM-5 diagnostic cluster severity score for diagnosis of PTSD thus providing only the provisional diagnosis.

## Conclusion

The problem density of PTSD after 10 months of earthquake is high among the adult survivors. Vulnerable groups for PTSD are elderly, females, illiterates and those injured in the earthquake. Though largely affected by earthquake, survivors are likely to use positive coping strategies. However, elderly and females employ more maladaptive coping strategies which further increase the risk for PTSD. The results of this study provide an empirical basis for acknowledging the existence of poor mental health among the adult survivors of earthquake suggesting the need for effective screening and promotion of positive coping strategies among the vulnerable groups. Study findings recommend executing further larger surveys in other settings for better generalization of the findings. Targeted preventative psychological and supportive interventional studies focusing on adaptive coping measures in the highly affected areas can be conducted.

## Abbreviations

DSM- 5: Diagnostic and Statistical Manual of Mental Disorders, 5th edition
PCL-5: PTSD checklist- according to DSM- 5 criteria
PTSD: Post traumatic Stress disorder
VDC: Village Development Committee
